# B Chromosomes in *Psalidodon scabripinnis* (Characiformes, Characidae) Species Complex

**DOI:** 10.3390/ani12172174

**Published:** 2022-08-25

**Authors:** Duílio M. Z. A. Silva, Jonathan P. Castro, Caio A. G. Goes, Ricardo Utsunomia, Mateus R. Vidal, Cristiano N. Nascimento, Lucas F. Lasmar, Fabilene G. Paim, Letícia B. Soares, Claudio Oliveira, Fábio Porto-Foresti, Roberto F. Artoni, Fausto Foresti

**Affiliations:** 1 Laboratory of Biology and Genetics of Fishes, Department of Structural and Functional Biology, Institute of Biosciences, São Paulo State University, Botucatu 18618-970, SP, Brazil; 2 Post-Graduate Program in Evolutionary Genetics and Molecular Biology, Department of Genetics and Evolution, Federal University of Sao Carlos, Sao Carlos 13565-905, SP, Brazil; 3 Laboratory of Evolutionary Genetics, Department of Structural, Molecular and Genetic Biology, State University of Ponta Grossa, Ponta Grossa 84030-900, PR, Brazil; 4 Laboratory of Fish Genetics, Department of Biological Sciences, Faculty of Sciences, São Paulo State University, Bauru 17033-360, SP, Brazil; 5 Laboratory of Fish Genetics, Department of Genetics, Institute of Biological Sciences and Health, Federal Rural University of Rio de Janeiro, Seropedica 23890-000, RJ, Brazil

**Keywords:** *Astyanax*, *Psalidodon*, supernumerary chromosome, gene expression, cytogenetics, repetitive DNA, meiosis

## Abstract

**Simple Summary:**

For more than a century, B chromosomes have been investigated in several eukaryotic species. These supernumerary genomic elements behave as parasites or provide fitness benefits to the hosts. They are mostly composed of repetitive DNA, but they also have protein-coding genes. B chromosomes are associated with differential gene expression and phenotypic effects. This makes them one of the most interesting genomic elements to investigate. Fish species of the *Psalidodon* genus harbor a great diversity of B chromosomes. Recent studies showed they share a common ancestor, persisting in the genus for a long time and enduring speciation processes. In the *Psalidodon scabripinnis* species complex, B chromosomes express their own genes, mostly related to cell cycle and gonad differentiation. Moreover, these B chromosomes are associated with functional effects, e.g., cell cycle extension. Here, we review the current knowledge regarding these elements in the *P. scabripinnis* species complex and propose a chromosome speciation model facilitated by the B chromosome manipulation of the cell machinery.

**Abstract:**

B chromosomes are extra-genomic components of cells found in individuals and in populations of some eukaryotic organisms. They have been described since the first observations of chromosomes, but several aspects of their biology remain enigmatic. Despite being present in hundreds of fungi, plants, and animal species, only a small number of B chromosomes have been investigated through high-throughput analyses, revealing the remarkable mechanisms employed by these elements to ensure their maintenance. Populations of the *Psalidodon scabripinnis* species complex exhibit great B chromosome diversity, making them a useful material for various analyses. In recent years, important aspects of their biology have been revealed. Here, we review these studies presenting a comprehensive view of the B chromosomes in the *P. scabripinnis* complex and a new hypothesis regarding the role of the B chromosome in the speciation process.

## 1. Introduction

Small extra fragments of genetic materials have been observed in the cells of some organisms since the first observations of chromosomes in the early 20th century. In most cases, these elements are lost [1], but some, denoted as B chromosomes, are maintained over generations. However, even after more than a century of research, many aspects of the biology of these enigmatic elements remain elusive.

B chromosomes can self-originate from the standard chromosomes of a species (intraspecific origin) [2,3] or from chromosomes of other species through hybridization or introgression events (interspecific origin) [4,5]. The source of the genetic material may originate from autosomes or sex chromosomes [6]. In some cases, their origin can be delimited to specific chromosomes [7,8,9,10]. However, in several species, the origin of the B chromosome cannot be determined due to the fast evolution of its sequences [11]. In most cases, B chromosomes are a mixture of DNA sequences acquired from several chromosomes of the standard genome [12,13,14] and/or organelles [15]. Most B chromosomes have a large repetitive DNA content, such as ribosomal DNA, satellite DNAs, U snRNA genes, histone genes, amplified telomeric sequences and transposable elements [6,16,17,18,19]. Even though these elements are frequently heterochromatic, young B chromosomes may show few repetitive DNA sequences, since they are euchromatic [7,20]. B chromosomes can also harbor protein-coding genes with various functions [13,14,21,22]. Among them, genes that may benefit the maintenance of B chromosomes and may be related to their evolutionary success have been detected [13,14,21]. Some of these genes are actively expressed and can even be translated into functional proteins [23,24,25,26], resulting in evident phenotypic effects [25,27].

In recent years, knowledge of B chromosomes has increased significantly with the application of powerful high-resolution technologies, such as third- and fourth-generation DNA and RNA sequencing. The recently discovered fascinating aspects of B chromosomes include the presence of an epistatically Y-dominant female sex determinant gene in cichlids [28]; the elimination of B chromosomes only in the roots of *Aegilops speltoides* during the embryonic stage [29]; the presence of a gene acquired by interspecific hybridization called haploidizer in *Nasonia vitripennis*, which causes the sexual conversion of females into males by expelling the entire genome coming from the sperm [26]; the paternal inheritance and escape of the B chromosome from elimination in male meiosis in mealybugs, in which the entire paternal genome is eliminated during gamete formation [30].

**Figure 1:**
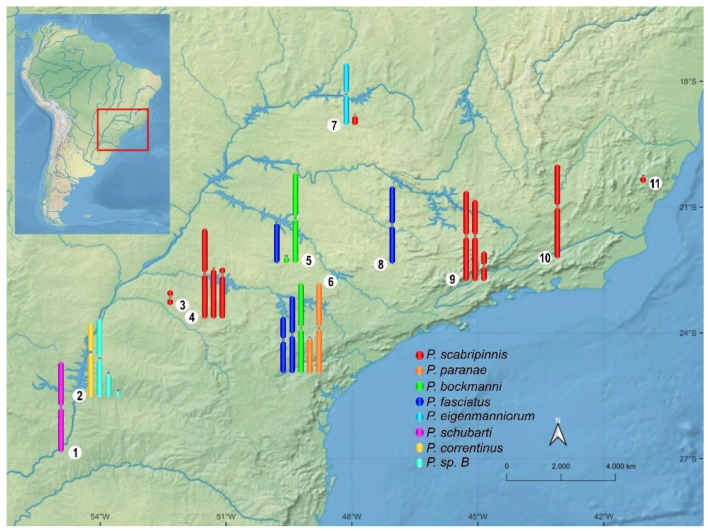
B chromosome diversity and distribution of *Psalidodon* species. 1—Missiones. 2—Foz do Iguaçu. 3—Maringá. 4—Marialva. 5—Bauru. 6—Botucatu. 7—Uberlândia. 8—Araras. 9—Campos do Jordão. 10—Pindamonhangaba. 11—Vitor Hugo. The distribution is based on Table S2. The figure shows the B chromosome variants found in the species from each region, not considering variants present in close streams.

**Figure 2:**
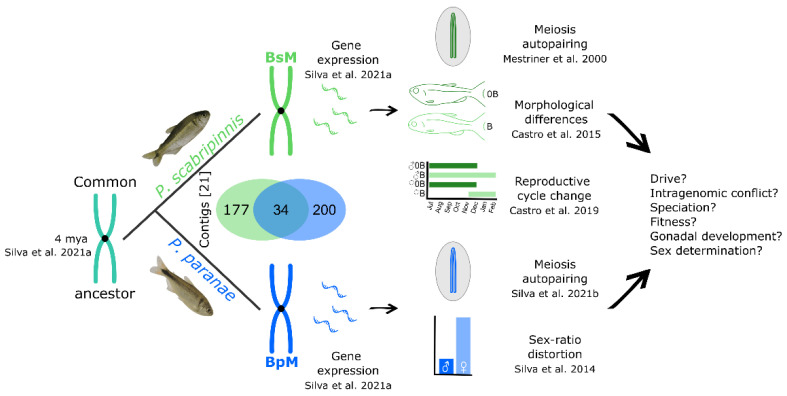
Origin, evolution, and effects of *Psalidodon scabripinnis* complex metacentric B chromosomes. Numbers in square brackets refer to references. On the right, features involving the B chromosomes that still need further investigation [8,21,38,39,40,41].

A strong diversity of B chromosomes is present in approximately 70 Neotropical fish species [31,32]. They can be euchromatic or heterochromatic, from micro to large B chromosomes [32,33]. However, in many cases, these elements are described with low population frequency or as being mitotically unstable and not uniformly present in all cells of the organism, hindering various types of analyses [32,33,34,35]. Characiformes species belonging to the *Psalidodon scabripinnis* complex [36] constitute excellent models for B chromosome studies, as they show populations with several B chromosome variants (Figure 1), some of which are mitotically stable and have varied frequencies, enabling a wide range of studies. In recent years, the B chromosomes of these species have been extensively investigated in several aspects (Table S1, Figure 2).

Recently, Terán et al. [37] recovered the putative monophyletic genus *Psalidodon* to include more than 30 species previously belonging to *Astyanax*. Before the re-division, the genus *Astyanax* included 11 species carrying B chromosomes with varying morphologies. Among them, the species that remained in the genus *Astyanax* carry only small acrocentric B chromosome variants (review in [21]). Therefore, the great diversity of B chromosomes previously described in *Astyanax* is now present in eight species of *Psalidodon* (Table S2, Figure 1).

We present a comprehensive review of the B chromosomes in two sister species belonging to the *P. scabripinnis* complex (*P. scabripinnis* and *Psalidodon paranae*), as well as perspectives for future studies, and propose a new hypothesis regarding the role of the B chromosomes in the speciation process.

## 2. Materials and Methods

We tried multiple databases as sources for this literature review: Google Scholar, PubMed, and Scopus using the descriptors: *Astyanax scabripinnis*, *Astyanax paranae*, *Psalidodon scabripinnis*, *Psalidodon paranae*, B chromosomes, and supernumerary chromosomes. The terms were combined in multiple ways using the Boolean term “AND”. The Google Scholar search showed the largest number of papers, including all the papers available in the other databases. In total, the Google Scholar search showed about 500 papers, among which 41 (Table S1) were selected for this review. We selected all the papers related to B chromosomes in the *Psalidodon scabripinnis* complex, without any discrimination regarding its presence/absence in specific databases, as we consider all of them useful to acknowledge.

Some relevant papers are not available on the web due to the time of publication. In these cases, we requested them from colleagues that maintain private libraries of scanned PDFs. This material can be requested from the corresponding authors.

We divided this review into topics aiming to provide a view of the B chromosome’s history in the *P. scabripinnis* complex, its transmission, structure, gene content, expression, and functional effects. Additionally, we provide two other topics, one proposing a new hypothesis about the role of B chromosomes in speciation and one proposing the *P. scabripinnis* complex as a model to study B chromosomes in fish. As the topics are interrelated, we chose this division based on the way they are encompassed in the greater part of published papers.

## 3. B Chromosomes in *Psalidodon scabripinnis* Complex

### 3.1. Origin

**Figure 3:**
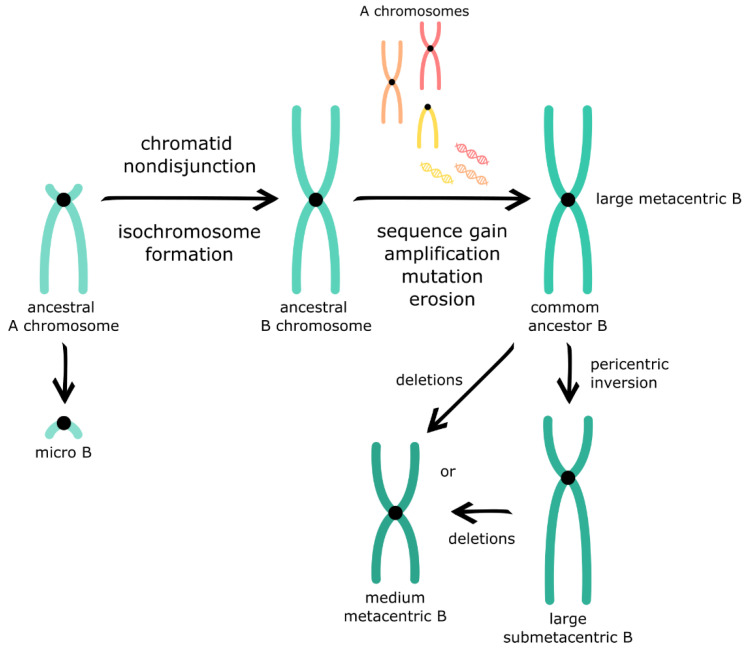
*Psalidodon* B chromosomes birth and evolution model.

In *Psalidodon*, the presence of B chromosomes has been widely recorded since its first description in *P. scabripinnis* [42]. *P. scabripinnis* constitutes a species complex with the greatest number of B chromosomes studied, harboring different morphologies from macro- to microchromosomes (Figure 1, Table S2). However, the large metacentric B chromosome (BM) variant with a similar size to the first autosomal pair is the most frequent in *P. scabripinnis* and in other species of *Psalidodon* (Figure 1, Supplementary Table S2). Based on this, Salvador and Moreira-Filho [42] hypothesized that this variant would have originated from the non-disjunction of a chromosome of the first autosomal pair. Later, Vicente et al. [43] described a BM variant in *P. scabripinnis,* in which heterochromatin blocks are restricted to the interstitial region of the two arms in a pattern that closely resembles the autosomal acrocentric 24th pair. Thus, the authors hypothesized that BM is an isochromosome originating from the long-arm sister chromatid non-disjunction of this pair (Figure 3). This hypothesis was later confirmed by Mestriner et al. [38] through molecular cytogenetic studies and analyses of chromosome pairing during meiosis, which will be discussed in the next section of this paper.

At the same time, Maistro et al. [44] observed contrasting R- and G-banding patterns between the BM chromosome and the first autosomal pair of *P. paranae*; therefore, if they originated from the first pair, they would have followed different evolutionary paths. Subsequently, by employing several banding techniques, such as C-banding, CMA_3_ staining, incorporation of 5-bromo-2′-deoxyuridine, and chromosome digestion with nine restriction endonucleases, Maistro et al. [45] reinforced the idea that this variant in this species could originate from an acrocentric pair by showing that the 21st and 22nd pairs share heterochromatin with the same compositional features as the B chromosome. This indicated that the BM variants in *P. scabripinnis* and *P. paranae* could have originated from the same ancestral acrocentric chromosome.

Both studies developed by Maistro et al. [44,45] analyzed samples from the Cascatinha stream, Botucatu, Brazil. First, they named the species *P. scabripinnis* [44] and later *P. scabripinnis paranae* [45], which was considered to be a subspecies of the *P. scabripinnis* complex; however, after the abolishment of this category, it was named *P. paranae* [46], the same as other populations of *P. paranae* from the Botucatu region. Mitochondrial DNA analyses revealed that the individuals of this population belong to a different species of the *P. scabripinnis* complex from the Campos do Jordão region analyzed by Salvador and Moreira-Filho, Vicente et al., and Mestriner et al. [38,42,43].

Despite BM variant predominance, the presence of several B chromosome variants within the same species is an intriguing point that raises several questions about the evolutionary dynamics of these chromosomes. Néo et al. [47] proposed that the BM and Bmicro (micro-B chromosome) variants may have originated simultaneously via centromere non-disjunction of the acrocentric 24th pair, followed by chromatid nondisjunction. However, in populations of *P. scabripinnis*, these variants were not found together, which would be expected if they had a simultaneous origin; thus, Moreira-Filho et al. [34] suggested an independent origin for both variants. Therefore, it is possible that the Bmicro and BM variants were not observed together in *P. scabripinnis* due to the low frequency of the Bmicro variant, as this variant occurs in only a few populations [34]. Furthermore, Néo et al. [47] proposed that other variants, such as BSM (large submetacentric B chromosome) and Bm, would have originated more recently from chromosomal rearrangements occurring on the B chromosome itself. Considering that the BSM variant is similar in size to BM, its origin could have been pericentric inversion, whereas the Bm variant could have originated from deletions of the BM or BSM variants. The low frequency of these variants observed by Ferro et al. [48] reinforces the idea that they originated recently. An alternative hypothesis is that both variants arose at the same time, but different B chromosomes were lost in different species/populations.

### 3.2. Predominance in Females

An interesting aspect of B chromosomes in *Psalidodon* is the predominance of BM in females. This pattern is observed even in BM variants with different C-heterochromatin patterns [34]. However, the reasons for this predominance are still unknown. In two cichlid fishes, the female-restricted B chromosomes are involved in the sex determination [27,28], but the molecular mechanisms involved are also a mystery.

Alternatively, Rocon-Stange and Almeida-Toledo [49] described a male-restricted Bmicro in a *P. scabripinnis* population, a similar scenario recently elucidated by multiple genomic approaches in *Astyanax mexicanus*, in which the authors showed a chromosomal drive for males and what they called supernumerary B-sex [50]. Thus, the mechanisms of the sex determination distortion pathways seem to be a frequent factor in these fish and are associated with the presence of B chromosomes, even in variants that follow different evolutionary paths. However, this remains an open question requiring further analysis, considering that the B chromosomes can predominate in males or females depending on the population analyzed.

We can also highlight the effects of B chromosome presence and seasonal variation between sexes. The pioneering study by Maistro et al. [51] revealed that the population of *P. paranae* from the Cascatinha stream has a predominance of BM in females (approximately 27% of the analyzed females) compared to males (100% of non-B carriers). Later, by reanalyzing the same population, Porto-Foresti et al. [52] observed an increase in BM frequency in females (57%) and the occurrence of this element in males (8.7%). Recently, Goes et al. [53] carried out a new survey, in addition to performing a comparative analysis between the data obtained from 2014 to 2017 and from 1994 to 1997 [51,52] with an interval of 20 years between the two samples. They verified an increase in the frequency of B chromosomes per individual in females (from 51% to 71%) and in males (from 7% to 31%). In males and females, the frequency of B chromosomes in the *P. paranae* population from the Cascatinha stream increased from 35% to 56% in the 20-year interval, indicating a B fixation in this population. Silva et al. [8] pointed out that in the *P. paranae* population from the Capivara River, Botucatu, Brazil, B chromosomes were present in 36.9% of females and only 3.7% of males, showing a clear bias towards a higher frequency in females. Vicente et al. [43] also reported a significantly higher frequency of B chromosomes in females in three populations of *P. scabripinnis* (the Pedras, Casquilho, and Perdizes streams, Campos do Jordão, Brazil), with 95.5%, 45.4%, and 50% of females carrying B chromosomes, respectively. The authors drew attention to the sex ratio bias in favor of females and its significant association with the occurrence of B chromosomes, with a highly disproportionate number of males lacking these chromosomes, corroborating the bias observed in other populations of *Psalidodon* harboring B chromosomes.

### 3.3. Geographic Variation

Porto-Foresti et al. [52] also showed different frequencies of B chromosomes in three stretches of the Cascatinha stream. The higher frequency in the first stretch was attributed to a genetic drift or an adaptive effect conferred by the presence of B chromosomes. Accordingly, Néo et al. [54] found that B chromosomes are present at high frequencies in two higher stretches of the Ribeirão Grande River, Campos do Jordão, Brazil, but absent in the lower stretch. The studies differed in sample size and altitude range. Néo et al. [54] analyzed 82.6 individuals per stretch on average and stretched at altitudes of 1920, 1800, and 700 m, whereas Porto-Foresti et al. [52] analyzed 21.6 individuals per stretch on average and stretched at altitudes of 880, 860, and 820 m. Despite these differences, in both studies, the B chromosome frequencies were higher in the headwaters.

These results are best explained by the parasitic theory [6]. Considering this theory, B chromosomes could be maintained by driving in the populations even though they might be harmful for B-carriers. Thus, the presence of B chromosomes could be more tolerated under favorable environmental conditions because the harmful effects would be best tolerated. As *P. scabripinnis* is best adapted to the headwaters of streams or small rivers [55], the populations inhabiting higher stretches probably occupy the most favorable environmental conditions, which makes them more tolerant to the presence of B chromosomes, whereas the lowest sites could have certain ecological conditions incompatible with the presence of harmful B chromosomes. Although no ecological analyses were performed, Néo et al. [54] highlighted two important ecological differences between the high- and low-altitude sites: (1) the reduced presence of potential predators in the high-altitude sites and (2) the lower species diversity at the high-altitude sites compared to the lower ones, which indicates a weaker level of resource competition in the first. Both differences were also observed between the Cascatinha stream stretches analyzed by Porto-Foresti et al. [52], in which only two species, *P. paranae* and *Phalloceros* sp., were observed inhabiting the first portion of the stream during decades of sampling, contrary to the greater diversity found in the lower stretches.

## 4. Transmission of B Chromosomes

The frequency of B chromosomes in natural populations is intrinsically correlated with the transmission of these elements to the offspring. In most cases, B chromosomes do not follow Mendelian laws of inheritance. They can be transmitted at rates higher than 0.5, which is called drive, and accumulate over generations. In contrast, transmission rates below 0.5 are also possible, leading to the disappearance of these elements over time [6]. In a pioneering study, Goes et al. [53] analyzed B chromosome inheritance patterns in *P. paranae*., revealing sex-dependent transmission. More specifically, this study revealed that female-inherited B chromosomes exhibit low rates of transmission to the offspring (kb = 0.15, on average), whereas those transmitted by males are close to neutrality (kb = 0.45) [53]. These results indicate the absence of a drive in the B chromosome variant in *P. paranae*. Despite this, the frequency of these elements has increased in the population (from the Cascatinha stream) in recent decades. This apparent contradiction suggests a possible mechanism of B chromosome elimination in the germline of *P. paranae* females and possible adaptive advantages to their carriers, as they increase in the population. Alternatively, this B chromosome could lose its capacity to accumulate after suffering an initial drive and reaching a maximum frequency supported by the population, in accordance with the parasitic theory (see section “B chromosomes in *Psalidodon*”). Individuals with two B chromosomes are very rare in the Cascatinha population [53,56], indicating that the fertilization between two gametes harboring B chromosomes is a rare event, or that the survival of 2B individuals is low. Both cases could be the result of the harmful effects of the B chromosome.

According to 3D cell analysis, the *P. scabripinnis* B chromosome occupies a peripheral position in the interphase nucleus [57], which seems to be common in some types of B chromosomes [58]. This peripheral territory is occupied by chromosomes that tend to be eliminated in hybrids [59] and other organisms [58]. Although 3D cell analysis has not been performed in studies of *P. paranae*, whole-chromosome painting experiments (with BM probes) mostly show 2D signs in the peripheral regions of the nucleus [8], which could be associated with elimination in female gametes. Clark and Akera [60] postulated that B chromosomes can achieve drive only through random positioning in dividing cells, as the mitotic spindle is asymmetric, and the B chromosome would always have more chances of going to the vegetative nucleus. However, if the B chromosome has a specific territory in the dividing cell, it could have a peculiar behavior, such as elimination. The peripheral position of B chromosomes in the nucleus is related to their heterochromatic content and activation status [57]. Thus, euchromatic B chromosomes in the early stages of evolution could occupy central regions in the nucleus, which could favor their transmission to germ cells, reaching the initial drive. Later, these B chromosomes could be modified, for example, via the acquisition of repetitive DNA sequences, becoming heterochromatic and inactivated. This new status could be responsible for moving them to peripheral positions, leading to their elimination, as postulated for the B chromosome of *P. paranae* [53].

This explanation does not consider the possible action of several genes involved in B chromosome transmission. For example, *nusap1* is present in the B chromosomes of four *Psalidodon* species, including *P. paranae* [21], and encodes a microtubule-associated protein [61]. The abnormal expression of this gene is associated with inappropriate mitotic spindle formation and cell-cycle dysregulation [62,63]. According to Akera et al. [64], both processes need to be altered to drive selfish elements. Thus, the B chromosomes of the *Psalidodon* species could benefit from the expression of this gene to obtain higher transmission rates in the early stages of evolution. Currently, this gene is highly amplified in these B chromosomes [21], which could result in their overexpression, leading to gamete malformation or B chromosome expulsion via the polar corpuscle.

Studies related to the transmission of B chromosomes in *P. paranae* present major challenges, such as: (1) the lack of knowledge about the reproductive behavior of the animals, as they are not model organisms in reproduction assays; (2) the annual breeding season—despite reports of split spawning in several *Psalidodon* species, the ideal reproductive period for the reproduction of animals in captivity is between the months of November and February, known as *piracema*; (3) the difficulty in handling—because *P. paranae* is not a model species, there are no stocks of domesticated brood stock. Thus, wild animals are collected close to the breeding season, but few can reproduce in captivity; (4) the difficulty in obtaining males with B chromosomes—despite an increase in males with B chromosomes in natural populations, as described above, they still represent a minority of individuals. Therefore, a targeted crossing that depends on males carrying the B chromosome is difficult.

Despite the abovementioned difficulties, assessing the detailed B chromosome transmission in *P*. *paranae* and *P*. *scabripinnis* is essential, mainly due to the possible elimination of these elements by females. B chromosomes are probably eliminated during the formation of the female gametes during the expulsion of the first or second polar bodies. Furthermore, only one population of *P. paranae* has known transmission patterns, making it necessary to compare these indices with populations that have different frequencies of B chromosomes. Finally, the low transmission rates described by Maistro et al. [53] contrasted with the maintenance of these elements in the population, making further experiments necessary to better understand the role of B chromosomes in the population.

## 5. Structure of B Chromosomes

The first studies with basic cytogenetic techniques on *Psalidodon* B chromosomes were limited to the morphology and patterns of C-heterochromatin. Subsequently, molecular cytogenetics provided the opportunity to analyze the genetic content of these elements using repetitive DNA probes. In a landmark study, Mestriner et al. [38] identified AT-rich and 51 bp-long satellite DNA in the genome of *P. scabripinnis*. This satellite, named As51, is mainly located in the distal heterochromatin regions of the acrocentric chromosomes of the standard complement and was found to be in the interstitial region in both arms of the BM chromosome. The almost entirely symmetrical distribution of this satellite in the arms of the BM and the self-pairing of this chromosome during meiosis provided a strong basis for the hypothesis that it originated from an isochromosome formation process, as proposed by Vicente et al. [43]. According to Mestriner et al. [38], the small distribution difference of this satellite between the two arms may be due to a pericentric inversion that would have moved a cluster from one arm to the other.

The 24th pair is one of the different chromosomes harboring the As51 satellite DNA, which was proposed by Vicente et al. [43] as an ancestor for the origin of the BM chromosome. However, Vicari et al. [65] showed that the BM chromosome of *P. scabripinnis* shares repetitive sequences with several other chromosomes in the A complement based on a map of C0t-1 probes. Therefore, it was not possible to precisely determine which chromosome pair the BM of *P. scabripinnis* originated from.

In a broader description of the molecular content of the B chromosome, Silva et al. [8,46,66] showed that H1 histone, 18S rDNA, and satellite DNAs are accumulated on this supernumerary chromosome. The 18S rDNA is in the distal portion of both arms in the BM and Bsm (medium submetacentric B chromosome) variants, unlike that observed in the BM of *P. scabripinnis*, in which 18S rDNA clusters are absent [65,67,68]. In addition, H1 histone genes are symmetrically distributed in the pericentromeric region of both arms of the BM variant of *P. paranae*. Interestingly, the Bsm variant presents a differential distribution of H1 between the arms, with a predominance of clusters in the long arm, which suggests the occurrence of a pericentric inversion with asymmetric breakpoints [8]. Both 18S rDNA and H1 histone genes are also co-located in the 2nd and 23rd pairs, which were delimited as the most parsimonious probable ancestors for the B chromosome in this species [8]. The 23rd pair is acrocentric and coincides with the origin proposed by Vicente et al. [43] for *P. scabripinnis*. Moreover, molecular analyses detected H3 histone sequences in the BM chromosome of *P. paranae*, but they were not visible by FISH [8].

As generally considered for satellite DNAs, the mapping of the microsatellites’ sequences (AC)_15_, (CAC)_10_, and (GA)_15_ indicates the high symmetry between the B-arms in *P. scabripinnis* [69], consistent with the isochromosome nature of this variant. Additionally, Silva et al. [66] showed the presence of microsatellite (AC)_15_ clusters located in the terminal region of both arms of the BM variant in *P. paranae*. Furthermore, in an extensive analysis based on satellite DNA mining by NGS and bioinformatics, Silva et al. [46] revealed the presence of 14 families of satDNA clustered in BM and distributed symmetrically. All repetitive DNA mapped in this B chromosome suggested that the B chromosome of *P. paranae* is also an isochromosome, which was later confirmed by meiotic studies showing their self-pairing [39], as revealed in the B chromosome of *P. scabripinnis* [38]. The higher content of repetitive DNAs in this B chromosome than in standard chromosomes constitutes the different heterochromatin patterns between them [45], indicating that B chromosomes follow their own evolutionary pathway.

The differences in repetitive DNA content between the B chromosomes of *P. scabripinnis* and *P. paranae* could indicate independent origins. However, the morphological similarities and identification of the same formation process point to a common origin. Repetitive DNA has a high evolutionary dynamic and can frequently colonize new chromosomes via different processes [70]. Thus, they represent a safe source for inferring chromosomal origins only in the case of recent events, in which the repetitive content of the involved chromosomes may be very similar and not have undergone many changes, as they also present different rates of evolution [8]. To overcome this issue, Silva et al. [21] performed high-throughput sequencing and bioinformatics analyses of protein-coding genes, corroborating the hypothesis of a common origin between the B chromosomes of *P. scabripinnis*, *P. paranae*, and other *Psalidodon* species.

## 6. B-Genes

The sister species *P. scabripinnis* and *P. paranae* have the presence of large metacentric B chromosomes, which share several protein-coding genes [21]. An in silico analysis showed that they harbor at least 211 and 234 sequences of coding regions, respectively, and 34 of these regions were common to both species. The analysis of the 27 genes corresponding to these regions showed the presence of 20 genes in the B chromosomes of *P. scabripinnis* and *P. paranae*, with 19 genes shared by both species and one specific gene unique to each one [21]. The presence of these genes on both B chromosomes cannot be explained by chance, reinforcing the hypothesis of their common origin (Figure 2). Furthermore, incomplete genes were identified on these B chromosomes with the same absent regions, which is more parsimoniously explained by remote common descent [21]. The authors further showed that the gene content of the B chromosomes of *P. scabripinnis*, *P. paranae*, *P. bockmanni*, and *P. fasciatus* is consistent with the phylogenetic relationships of these species, indicating that their B chromosomes appeared in a common ancestor around 4 mya.

Some of the incomplete genes identified on the B chromosomes of *P. scabripinnis* and *P. paranae* may have originated from pseudogenization, as it seems to occur in the case of the gene *amhr2*, which appears to be a processed pseudogene [21]. The pseudogenization of the B chromosome could involve several processes, such as retrotransposition, invasion by transposable elements, formation of satellite DNA sequences, and gene erosion [12,71] (Figure 3). Thus, a thorough investigation of the B-pseudogenes structure may clarify the evolutionary processes that shape their current configuration.

A genomic analysis of the B chromosomes of *P. scabripinnis* and *P. paranae* revealed that among the 21 genes detected, eight codes for functions relate to the cell cycle and gametogenesis, and if these genes are functional at the right place and time, they may influence crucial processes in B chromosome transmission and persistence [21]. Their expression patterns are discussed in the next section of this paper.

In addition, H1 and H3 histone coding repetitive sequences on the B chromosome of *P. paranae* have been described [8]. An analysis of these genes revealed that histone H1 sequences have alterations in 6 of the 150 amino acids analyzed, whereas histone H3 sequences showed no variation. An analysis of the synonymous and non-synonymous substitutions indicated that the purifying selection may be relaxed for the histone H1 sequences present on the B chromosome, as expected if these sequences are inactive; this may explain their differential amplification on the B chromosome, as verified by FISH [8]. In addition, most of the H1 and H3 histone sequences obtained from this B chromosome have the same putative amino acid sequence as those obtained from 0B genomic DNA, revealing that they are potentially active.

## 7. Expression and Effects of B-Genes

For a long time, it was agreed that B chromosomes, found in several eukaryotes, were non-functional and practically devoid of genes, or without essential genes [6,72,73]. However, the advancement of molecular techniques, especially next-generation sequencing, has made it possible to access the genome and transcriptome of species with B chromosomes, leading to the discovery of functional evidence associated with the presence of these elements in several organisms.

Recent studies performed on *Psalidodon* indicated differential gene expression in individuals carrying B chromosomes [21,39,40,74]. A transcriptome analysis showed a high expression of two paralog genes on the B chromosome related to the cell cycle (*nobox* and *msh4*) in females [21]. The differential expression of sex-determining genes was also identified in individuals carrying the B chromosome in *P. scabripinnis*. B-carrying males showed a high expression of the gene related to sex determination and testicular differentiation *dmrt1* [74], whereas females with the B chromosome showed atypical expression of the ovarian development gene *foxl2a* [40]. Considering that these genes are not present on the B chromosome, these expression profiles suggest that genes other than *nobox* and *msh4* may be involved in the sex determination gene cascade, which, in turn, alters the expression patterns of the sexual genes *dmrt1* and *foxl2a* [75,76].

On the other hand, the accumulation of 5-mC signals on the B chromosome of *P. scabripinnis* suggests that it could contain silenced regions, which could be the result of the association of different types of repetitive DNA sequences in their arms, as with the transposable elements Tc1 Mariner and LINE [77] the As51 satellite DNA [78].

A phenotypic effect of B chromosomes in *P. scabripinnis* is the alteration of the reproductive cycle of B-carrying individuals. Studies have indicated that females without B chromosomes have a longer reproductive cycle than females with B chromosomes, which have a shorter but later cycle [79]. This finding, when analyzed against upregulated *dmrt1* expression and extended spermatogenesis in males carrying B chromosomes, may explain the seasonal dynamics and the process involved in the maintenance of this chromosome in the population [40,74]. Another phenotypic effect of the B chromosomes can be found in populations of *P. scabripinnis*, in which the 1B individuals have morphological alterations in body shape [80].

## 8. Meiotic Behavior of B Chromosomes

B chromosomes exhibit peculiar meiotic behaviors. These elements form univalents in meiotic cells with a single B chromosome and iso-B chromosome self-pairs. In cells with more than one B chromosome, these elements can form different structures, such as univalent, bivalent, and trivalent structures [81,82,83,84].

The large metacentric B chromosome variants of *P. scabripinnis* and *P. paranae* are isochromosomes that self-pair during meiosis [8,38,39]. Furthermore, when meiotic cells of *P. paranae* have two iso-B chromosomes, they self-pair and optionally pair with each other [39]. This ability may confer an adaptive advantage to these elements to cross-checkpoints during the cell cycle and succeed through divisions. By doing this, the B chromosomes of *P. scabripinnis* and *P. paranae* can escape the meiotic silencing of unsynapsed chromatin processes and express their own genes [21], and if they are related to the meiosis process, they may help the transmission of B chromosomes. This is the case for the meiosis-specific gene *msh4*, which is expressed from B chromosomes in the ovaries of both species [21,39] and is related to the meiotic recombination and proper segregation of homologous chromosomes [85,86].

The self-pairing process of B chromosomes is responsible for the homogenization of their sequences [83], which results in the high sequence conservation of some genes present in B chromosomes, as demonstrated by Silva et al. [21]. As these genes may be essential for the adaptive success of B chromosomes, their conservation leads to the possible expression of functional proteins. In maize, many sequences present on B chromosomes are undergoing intense degeneration processes, as these elements are nonessential and have a relaxed purifying selection [11]. However, the sequences that might be useful are conserved. In this sense, the *msh4* gene has copies with complete coding regions on the B chromosomes of *P. scabripinnis* and *P. paranae*, although some of them have some non-synonymous substitutions, implying that it may be an important gene for the maintenance of B chromosomes in these species [21].

## 9. B Chromosome and Speciation

B chromosomes are present in several cryptic species of *Psalidodon* (Table S2), including those belonging to the *P. scabripinnis* complex. In some cases, incipient speciation processes are related to the existence of B chromosomes in *P. scabripinnis* [41,80], pointing out the possible involvement of Bs in their speciation processes. Here, we propose a hypothesis about how the B chromosomes could act, facilitating the fixation of chromosomal alterations, such as inversions [87]. By this mechanism, the B chromosome influence could lead to the formation of reproductive barriers even in sympatric populations, thus generating new species. Several findings in the *Psalidodon* genus discussed below underpin this hypothesis and it would be interesting to assess whether similar mechanisms could be present in other groups.

Regarding allopatric speciation, Castro et al. [80] showed evidence of incipient speciation between two populations of *P. scabripinnis* from the Ribeirão Grande River isolated by a waterfall with over 1000 m depth. A reproductive and molecular analysis suggested the pre-zygotic reproductive isolation and absence of gene flow between them. Their karyotypes were identical, except for the presence of a B chromosome only in the population of the higher altitude. This indicates that allopatric speciation and chromosomal differences result from the evolutionary processes that occurred during the isolation period. Similarly, Castro et al. [41] demonstrated the *P. scabripinnis* populations differentiation in the Atlantic Forest through geometric morphometry, cytogenetic markers, induced breeding, and phylogenetic inferences, reinforcing the occurrence of allopatric cryptic species.

On the other hand, *P. scabripinnis* populations presenting different karyotypes without intermediates hybrids were found in sympatry and syntopy, highlighting a probable sympatric speciation. The *P. scabripinnis* population from São Francisco River showed two cytotypes with 2*n* = 50 (cytotype I) and 2*n* = 48 (cytotype II) chromosomes and distinct karyotypic formulae without intermediate hybrids [88]. Individuals with cytotype II also had a heteromorphic pair and an acrocentric B chromosome in some cells. The authors could not precisely indicate whether the chromosomes of the heteromorphic pair were B chromosomes. In another population from the Tatupeba stream, Fernandes and Martins-Santos [89] observed three sympatric cytotypes with different diploid numbers (2*n* = 50, 2*n* = 48, and 2*n* = 46) and harboring different types of B chromosomes. Additionally, Castro et al. [41] showed that the individuals of this population with 2*n* = 50 and 2*n* = 48 are morphologically different. In both cases, B chromosomes are related to chromosomal rearrangements that differentiate probable cryptic species occurring in sympatry.

Limeira et al. [90] showed that individuals with B chromosomes are genetically differentiated from 0B individuals in the population of the Lavrinha stream: 0B individuals have private alleles and the B-harboring subpopulation have monomorphic locus and heterozygosity excess. They also demonstrated a recent bottleneck largely driven by the B-harboring subpopulation. This indicates that the B-harboring subpopulation can belong to a differentiating group.

Considering the B chromosomes association with allopatric and sympatric speciation, an important question regarding the role of this genomic element in these processes remains: could B chromosomes be directly involved in the process of speciation, or do they just follow the evolutionary species history? The pachytene checkpoint model of speciation proposed by Foe [87] predicts that the pachytene checkpoint can lead to new species formation, even within a freely interbreeding population. The pachytene checkpoint compares paired homolog chromosomes during meiosis eliminating cells with unmatched chromosome pairs. This improper pairing may be the result of different chromosomal rearrangements, including chromosomal inversions, that will decrease the recombination rate in and around the inverted segment, leading to genetic differentiation [87]. Unless the pachytene checkpoint process is 100% efficient, some gametes with inverted chromosomes can be produced. If these inversions capture enough adaptative alleles, they can be fixed in a subpopulation [87]. The pachytene checkpoint can act as a barrier to the gene exchange between them, leading to new species formation, even in sympatry. In this case, the pachytene checkpoint would act like geographical isolation in allopatric speciation.

Pachytene checkpoint surveillance is related to the crossover development [91]. These structures are formed after the homologous recognition and pairing during meiosis initiated with the formation of double-strand breaks (DSBs) in the DNA, followed by their repairing using the homolog DNA as a template. Among the genes regulating these processes, *msh4* is expressed from the B chromosomes of *P. scabripinnis* and *P. paranae*, resulting in an overexpression in ovaries and testes [21,39]. The Msh4 protein belongs to the mismatch repair protein family [92]. The Msh4 protein’s role is strictly related to the pachytene checkpoint, as unrepaired DSBs or unresolved recombination intermediates activate the pachytene checkpoint system [93]. The overexpression of the *msh4* gene in 1B individuals may indicate the ability of these chromosomes to manipulate the pachytene checkpoint for their own benefit. In addition, other B-genes related to the cell cycle may be involved in this manipulation.

**Figure 4:**
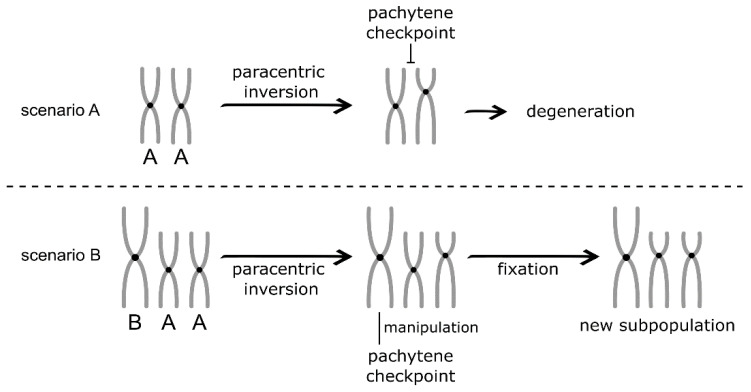
Pachytene checkpoint speciation model facilitated by regulatory networks impacted by the B chromosome. In scenario A, the pachytene checkpoint blocks the fixation of rearranged chromosomes. In scenario B, the manipulation of pachytene checkpoint by the B chromosome for its own correct segregation allows rearranged chromosome to be fixed in a new subpopulation.

Thus, we speculate that B chromosomes and their functional effects could surpass the meiosis checkpoints in a parasite-like manner, facilitating their own peculiar segregation and perpetuation in the populations. As a side effect, this manipulation could allow the rearranged chromosomes to complete the division. For this reason, B chromosomes could actively facilitate the fixation of inversions in subpopulations, leading to the rise of new karyotype variants and, possibly, to a speciation process (Figure 4). Several groups showing B chromosomes with complex phylogeny and cryptic species make this hypothesis possible to test.

Moreover, intragenomic conflict elements such as transposable elements, imprinted genes, and meiotic drivers are implicated in speciation [94]. These elements can re-shape regulatory pathways and the karyotype evolution during the arms race against the genome, creating enhancers and suppressors of drive leading to speciation. Keeping in mind that the B chromosomes have large blocks of genome duplications and several kinds of selfish DNA, they are probable spots for these new interactions. As sex chromosomes that evolve faster than autosomes [95], B chromosome evolution and interactions with A genomes can rapidly create incompatibilities between two isolated subpopulations.

Several studies have shown that these chromosomes can play different biological roles such as sex determination, adaptative advantages, and can even be deleterious. Here, we propose a new role for the B chromosomes in the *P. scabripinnis* complex. As a byproduct of their selfish behavior, these chromosomes might have played an important role in the speciation process and diversification of the *P. scabripinnis* group. Although this hypothesis still requires testing in further studies, one must say that the *P. scabripinnis* complex constitutes a good model.

## 10. *Psalidodon scabripinnis* as a Study Model

The *P. scabripinnis* complex has potential as a model organism for the analysis of the biology of B chromosomes. Several aspects of the *P. scabripinnis* B chromosomes have already been discovered, such as their composition, chromatin structure, transmission rate, epigenetic influence, phenotypic effects, and evolutionary history. Although the information available today has led to significant advances, these elements remain enigmatic. Currently, high-resolution techniques are available and considered very helpful for the advancement and exploration of this field. For example, the use of high-resolution genome assembly can help reveal the entire B chromosome structure and elucidate the complex structures formed by repetitive elements and duplications along these element sequences [96].

Directed crosses open up the opportunity to perform functional studies on B-genes. As an example, Imarazene et al. [50] generated *gdf6b* knockout mutants of *A. mexicanus* to prove this gene to be a master-sex gene using the CRISPR/Cas9 method. This gene has a specific copy on the B-sex chromosome, promoting male sex determination in this species [50]. However, the laboratory reproduction of the *P. scabripinnis* complex species is still a challenge to overcome.

Recently, cell lines were obtained from cell cultures of *P. paranae* with and without B chromosomes at the Laboratory of Fish Biology and Genetics, Botucatu, Brazil, as well as from *P. scabripinnis* by the Laboratory of Evolutionary Genetics, Ponta-Grossa, Brazil [57]. These cell lines constitute an important material for cytogenetic, molecular, and functional studies. In cytogenetic studies, the use of cell lines simplifies techniques, such as chromosome microdissection, chromosome painting, and single-copy gene mapping by special FISH techniques, such as CARD-FISH (catalyzed reporter deposition FISH), to provide a clean material with few cellular debris.

In addition, the cell culture technique has shown potential to be applied in the transfection of exogenous DNA, the selection of promoters, and gene knockout, and has already been applied to different species, such as salmon, medaka, and carp [97,98,99,100,101]. Additionally, using fibroblast cell culture, Trifonov et al. [102] showed the transcription of a B-specific protein-coding sequence in the Siberian roe deer. The abovementioned studies showed that cell cultures used in the study of B chromosomes could help elucidate B-gene expression at the RNA and protein levels, as well as their function, activity, and interactions.

## 11. Conclusions

Tremendous effort has been made over the years to clarify the biology of B chromosomes of the *P. scabripinnis* complex species and *Psalidodon* in general. Nonetheless, some aspects of these genomic elements remain unexplored, such as their relationship with microRNAs, long non-coding RNAs, transposable elements, and protein expression, which constitute an extensive field to be explored. Finally, the study of the phenotypes associated with the presence of B chromosomes, as well as the functional studies using genetic manipulation, may provide interesting answers regarding these enigmatic genomic elements.

## Data Availability

Not applicable.

## Supplementary Materials

The following supporting information can be downloaded at: https://www.mdpi.com/article/10.3390/ani12172174/s1, References [103,104,105,106,107,108,109,110,111,112,113,114,115,116,117] are cited in the supplementary materials. Table S1: Summary of B chromosome studies in *P. scabripinnis* species complex.; Table S2: Diversity of B chromosomes in *Psalidodon* genus.
