# Supplementary material for: B Chromosomes in Psalidodon scabripinnis (Characiformes, Characidae) Species Complex
Source: Animals (Basel). 2022 Aug 25;12(17):2174. doi: 10.3390/ani12172174 (PMC9454733; doi:10.3390/ani12172174)
Supplement: Supplementary file 1 [file animals-12-02174-s001.zip › animals-1856544-supplementary.pdf]

Table S1. Summary of B chromosome studies in *P. scabripinnis* species complex.

Species	Methodology	Population	Reference	Year
P. scabripinnis, P. paranae	C, GE, MG, NGS	Cascatinha River	Silva et al. [21]	2021
P. scabripinnis	C, MC, Ep	Lavrinha stream	Barbosa et al. [76]	2021
P. scabripinnis	C, MC, ND	Lavrinha stream	Schemczssen-Graeff et al. [54]	2020
P. scabripinnis	C, MC, GE, RC	Lavrinha stream	Castro et al. [73]	2019
P. scabripinnis	MG	Lavrinha stream	Limeira et al. [90]	2019
P. scabripinnis	MC, GE	Lavrinha stream	Castro et al. [72]	2019
P. scabripinnis	C, MC, SR	Lavrinha stream	Cornelio et al. [78]	2017
P. scabripinnis	C, MC	Lavrinha stream, Lake of Pedalinho, Perdizes stream	Barbosa et al. [66]	2017
P. scabripinnis	C	São Francisco River	Klassmann and Martins-Santos [88]	2017
P. scabripinnis	C, MC	Lavrinha stream, Ribeirao Grande stream, Tatupeba stream	Barbosa et al. [77]	2015
P. scabripinnis	C, MC, MO	Ribeirão Grande stream	Castro et al. [79]	2014
P. scabripinnis	C, MC, M	Pedra's stream	Vicari et al. [62]	2011
P. scabripinnis	C	Tatupeba stream	Fernandes and Martins-Santos [89]	2005
P. scabripinnis	Review	Review	Moreira-Filho et al. [34]	2004
P. scabripinnis	C, MC	Lavrinha stream, Capivari stream, Carpas lake, Fojo stream	Ferro et al. [45]	2003
P. scabripinnis	C	São Domingos stream	Alves and Martins-Santos [103]	2002
P. scabripinnis	C	Jataí stream	Araújo and Morelli [104]	2000
P. scabripinnis	C, MC, M	Pedra's stream	Mestriner et al. [40]	2000
P. scabripinnis	C, GV	Ribeirão Grande stream	Néo et al. [44]	2000
P. scabripinnis	C, GV	Ribeirão Grande stream	Néo et al. [51]	2000
P. scabripinnis	C	Yukatan stream	Mizoguchi and Martins-Santos [105]	1997
P. scabripinnis	C	Piracuama stream	Souza and Moreira-Filho [106]	1995
P. scabripinnis	C	Córrego das Pedras	Fauaz et al. [107]	1994
P. scabripinnis	C, SR	Jucu River	Rocon-Stange and Almeida Toledo [46]	1993
P. scabripinnis	C	Córrego das Pedras	Salvador and Moreira-Filho [38]	1992
P. paranae	T, D	Cascatinha River	Goes et al. [50]	2021
P. paranae	MC, MG	Cascatinha River	Goes et al. [108]	2021
P. paranae	MC, M, GE	Cascatinha River	Silva et al. [67]	2021

P. paranae	MC	Capivara River	Silva et al. [43]	2017
P. paranae	C, MC, MG	Capivara River	Silva et al. [63]	2016
P. paranae	C, MC, MG	Capivara River	Silva et al. [8]	2014
P. paranae	C, MC	Tagaçaba stream	Abelini et al. [109]	2014
P. paranae	C	Cascatinha River	Santos et al., [53]	2012
P. paranae	C	Cascatinha River	Maistro et al. [42]	2000
P. paranae	C	Cascatinha River	Maistro et al. [41]	1999
P. paranae	C, GV	Cascatinha River	Porto-Foresti et al. [49]	1997
P. paranae	C	Cascatinha River	Maistro et al. [48]	1994
P. paranae	C	Araquá River	Maistro et al. [110]	1994
P. paranae	C	Araquá River	Maistro et al. [111]	1992

C = cytogenetics, GE = gene expression, MG = molecular genetics, NGS = next generation sequencing, MC = molecular cytogenetics, Ep = epigenetics, ND = nuclear dynamic, RC = reproductive cycle, SR = sex-ratio, MO = morphometry, M = meiosis, GV = geographic variation, T = transmission, D = dynamics.

Table S2. Diversity of B chromosomes in *Psalidodon* genus.

**Species**	**Population**	**River Basin**	**County**	**2n**	**Bs**	**Size**	**Shape**	**Reference**
P. bockmanni	Água da Madalena stream	Paranapanema River	Botucatu, BRA	50	0-1	large	m	Silva et al. [63]
P. bockmanni	Alambari River	Paranapanema River	Bauru, BRA	50	0-1	micro	a	Hashimoto et al. [112]
P. bockmanni	Alambari River	Paranapanema River	Bauru, BRA	50	0-1	large	m	Daniel et al. [113]
P. correntinus	Iguaçu River	Paraná River	Foz do Iguaçu, BRA	36	0-1	large	sm	Ahmad et al. [114]
P. eigenmanniorum	Caetano Stream	Paraíba River	Uberlândia, BRA	48	0-2	large	m	Torres-Mariano and Morelli [115]
P. fasciatus	Água da Madalena stream	Paranapanema River	Botucatu, BRA	45-48	0-1	large	m	Silva et al. [63]
P. fasciatus	Água da Madalena stream	Paranapanema River	Botucatu, BRA	45-48	0-1	medium	sm	Silva et al. [63]
P. fasciatus	Alambari River	Paranapanema River	Bauru, BRA	46	0-1	medium	a	Hashimoto et al. [112]
P. fasciatus	Araras stream	Paranapanema River	Araras, BRA	48	0-1	large	m	Silva et al. [63]
P. paranae	Araquá River	Tietê River	Botucatu, BRA	50	0-1	large	m	Maistro et al. [110]
P. paranae	Araquá River	Tietê River	Botucatu, BRA	50	0-1	large	m	Maistro et al. [111]
P. paranae	Capivara River	Tietê River	Botucatu, BRA	50	0-1	macro	m/sm	Silva et al. [8]

P. paranae	Cascatinha River	Tietê River	Botucatu, BRA	50	0-2	large	m	Maistro et al. [48]
P. paranae	Cascatinha River	Tietê River	Botucatu, BRA	50	0-1	medium	a	Santos et al. [53]
P. paranae	Tagaçaba Stream	Ivaí River	Maringá, BRA	50	0-1	large	m	Abelini et al. [109]
P. scabripinnis	Lavrinha stream	Paraíba do Sul River	Campos do Jordão, BRA	50	0-1	large	m/sm	Ferro et al. [45]
P. scabripinnis	Capivari stream	Sapucai-Guaçu River	Campos do Jordão, BRA	50	0-1	large	m	Ferro et al. [45]
P. scabripinnis	Carpas lake	Sapucai-Guaçu River	Campos do Jordão, BRA	50	0-1	large	m/sm	Ferro et al. [45]
P. scabripinnis	Fojo stream	Sapucai-Guaçu River	Campos do Jordão, BRA	50	0-1	large	m/sm	Ferro et al. [45]
P. scabripinnis	Fojo stream	Sapucai-Guaçu River	Campos do Jordão, BRA	50	0-2	medium	m	Ferro et al. [45]
P. scabripinnis	Ribeirão Grande stream	Paraíba do Sul River	Campos do Jordão, BRA	50	0-2	large	m/sm	Néo et al. [44]
P. scabripinnis	Ribeirão Grande stream	Paraíba do Sul River	Campos do Jordão, BRA	50	0-1	medium	m	Néo et al. [44]
`*P. scabripinnis*	Jataí stream	Paranaíba River	Uberlândia, BRA	50	0-1	small	a	Araújo and Morelli [104]
P. scabripinnis	Jucu River	Jucu River	Vitor Hugo, BRA	50	0-4	micro	a	Rocon-Stange and Almeida Toledo [46]
P. scabripinnis	Pedra's stream	Sapucaí river	Campos do Jordão, BRA	50	0-2	large	m	Salvador and Moreira-Filho [38]
P. scabripinnis	Piracuama stream	Paraíba do Sul River	Pindamonhangaba	50	0-1	large	m	Souza and Moreira-Filho [106]
P. scabripinnis	São Domingos stream	Ivaí River	Maringá, BRA	48	0-1	small	m	Alves and Martins-Santos [103]
P. scabripinnis	Tatupeba stream	Ivaí River	Marialva, BRA	50	0-1	large	m	Fernandes and Martins-Santos [64]
P. scabripinnis	Tatupeba stream	Ivaí River	Marialva, BRA	46	0-1	small	a	Fernandes and Martins-Santos [64]
P. scabripinnis	Tatupeba stream	Ivaí River	Marialva, BRA	48	0-1	medium	st/a	Fernandes and Martins-Santos [64]
P. scabripinnis	Yukatan stream	Ivaí River	Marialva, BRA	50	0-1	large	m	Mizoguchi and Martins-Santos [105]
P. schubarti	Paraná River	Paraná River	Missiones, ARG	36	0-1	large	m	Moreira-Filho et al. [116]
P. sp. B	Salto Caxias reservoir, Iguaçu River	Paraná River	Foz do Iguaçu, BRA	50	1-2	large	m	Fazoli et al. [117]
P. sp. B	Salto Caxias reservoir, Iguaçu River	Paraná River	Foz do Iguaçu, BRA	50	1-2	small	sm	Fazoli et al. [117]
P. sp. B	Salto Caxias reservoir, Iguaçu River	Paraná River	Foz do Iguaçu, BRA	50	1-2	micro	a	Fazoli et al. [117]

2n = diploid chromosome number. Bs = number of B chromosomes. a = acrocentric. m = metacentric. sm = submetacentric. st = subtelocentric.
